# Structural characterization and antiviral activity of polysaccharide from *Codonopsis pilosula*

**DOI:** 10.1007/s13659-026-00649-5

**Published:** 2026-10-01

**Authors:** Ya-hui Song, Fang-qin Mao, Meng-fan Liu, Zhang-quan Liu, Qing-yan Xin, Lan Yu

**Affiliations:** School of Pharmacy, Zunyi Medical University, Zunyi, 563000 Guizhou China

**Keywords:** Polysaccharide, *Codonopsis pilosula*, Structure characterization, Antiviral

## Abstract

Codonopsis polysaccharides exhibit diverse biological activities, but their antiviral effects against the H1N1 influenza A virus and how these activities change after sulfation modification remains unclear. In this study, crude Codonopsis polysaccharide (COP) was obtained via water extraction and alcohol precipitation, and COP-1 (2.1 × 103 Da, β-D-(2 → 1)-fructan) was isolated and purified. Both COP and COP-1 were modified by sulfation using the sulfur trioxide-pyridine method, yielding derivatives SCOP and SCOP-1 with degrees of substitution of 1.792 and 1.403, respectively. Antiviral activity was evaluated in MDCK cells using MTT and TCID₅₀ assays, revealing that COP, SCOP, and SCOP-1 all inhibited H1N1 replication, with sulfation significantly enhancing antiviral activity. Time-of-addition experiments indicated that inhibition occurred during the early phase of infection (0–4 h). Annexin V-PI staining confirmed that these compounds reduced virus-induced early apoptosis. In H1N1-infected A549 cells, they suppressed NF-κB p65 phosphorylation and downregulated the expression of *IL-6, IL-8, MCP-1, CCL-5, TNF-α*, and *TRAIL*. Overall, Codonopsis polysaccharides and their sulfated derivatives exert anti-influenza effects through a triple mechanism involving inhibition of early viral replication, anti-apoptotic activity, and anti-inflammatory action, making them promising candidates for antiflu therapeutics.

**Graphical Abstract:**

**Figure:**
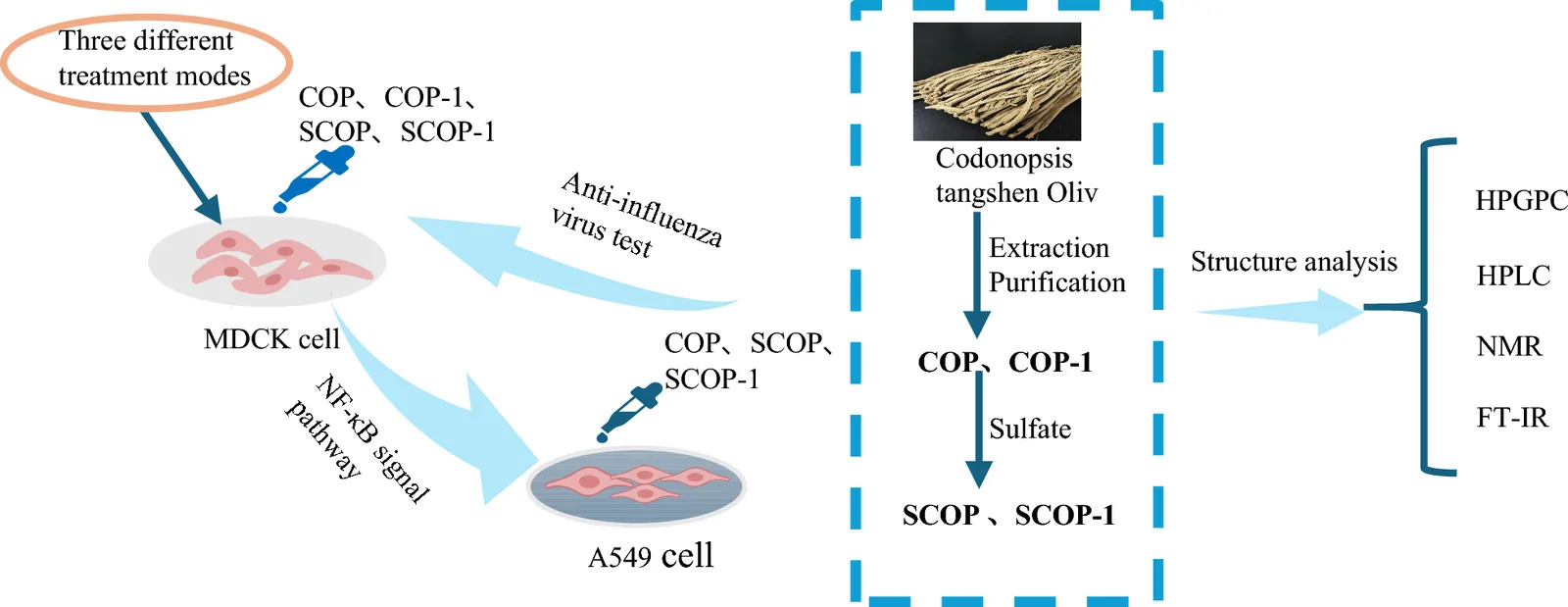

**Supplementary Information:**

The online version contains supplementary material available at https://doi.org/10.1007/s13659-026-00649-5.

## Introduction

*Codonopsis pilosula (Franch.) Nannf.,* a perennial herb of the family Campanulaceae, is a pharmacologically important traditional medicinal plant widely used in Traditional Chinese Medicine (TCM) as a spleen- and lung-tonifying agent. Pharmacological and clinical evidence supports its efficacy in enhancing spleen and lung function, augmenting hematopoiesis, and promoting body fluid generation [30]. As a functional food, *C. pilosula* is commonly incorporated into medicinal decoctions, soups, wines, and porridges [28]. Codonopsis polysaccharides (COP) are its core bioactive components, exhibiting diverse pharmacological activities including antioxidant [49], antitumor [38], hypoglycemic, immunomodulatory [11], and antiviral effects [31]. The antiviral mechanisms of plant polysaccharides involve direct inhibition of viral adsorption and replication, as well as indirect enhancement of host antiviral immunity [37]. Notably, the biological activities of polysaccharides are closely associated with their molecular structures [7,36]. Sulfation modification, which introduces negatively charged sulfate groups to alter molecular conformation, can enhance interactions with enveloped viruses and thereby improve antiviral activity [27]. Sulfated polysaccharides are an important class of semi-synthetic derivatives. The density and position of sulfate groups directly influence their antiviral efficacy, and they have been shown to exert broad-spectrum inhibitory effects against various enveloped viruses, including influenza virus, human immunodeficiency virus (HIV), and herpes simplex virus (HSV) [8,34].

Influenza, caused by the influenza virus (IV), is a global acute respiratory infectious disease characterized by seasonal annual epidemics and sporadic pandemics, and remains a priority for global public health [12]. The influenza virus (IV) belongs to the family Orthomyxoviridae and is an enveloped virus possessing a segmented, single-stranded, negative-sense RNA genome [39]. Based on antigenic differences in the nucleoprotein (NP), influenza viruses are classified into three types: A, B, and C. Among these, the H1N1 subtype of influenza A virus (IAV) has attracted particular attention due to its high pathogenicity [35]. Current antiviral agents, including neuraminidase inhibitors and M2 ion channel blockers [32], are increasingly limited by drug resistance and associated toxicities [26,43], highlighting the urgent need for alternative therapeutics with improved safety profiles [21,24]. Natural polysaccharides represent a promising avenue for such drug discovery.

Building upon our previous structural elucidation of the homogeneous polysaccharide COP-1 from *C. pilosula* [25], this study further investigated the in vitro anti-influenza activities of COP-1 and its sulfated derivatives SCOP and SCOP-1. COP-1 was isolated, purified, and structurally characterized. Antiviral efficacy was evaluated using an H1N1 influenza virus infection model in MDCK cells, while anti-inflammatory effects and underlying molecular mechanisms were explored in A549 cells through analysis of inflammatory cytokine expression. These findings aim to provide experimental evidence and theoretical support for the development of novel natural anti-influenza agents.

## Material and methods

### Materials

*Codonopsis pilosula* was purchased from Daozhen County, Guizhou, China, and authenticated by Professor Yang Jianwen of the Department of Pharmacognosy, Zunyi Medical University. All common chemical reagents used in this study were of analytical grade. Pullulan standards (analytical grade, for GPC) were purchased from Sigma-Aldrich (USA). Influenza A virus (IAV, H1N1), MDCK cells, and A549 cells were provided by the Department of Microbiology, Zunyi Medical University. Phosphate-buffered saline (PBS) and MTT were obtained from Beijing Solarbio Science & Technology Co., Ltd. (China). DMEM high-glucose medium, fetal bovine serum, 0.25% trypsin‑EDTA, 0.25% trypsin, and penicillin‑streptomycin were purchased from Gibco (USA). All cells were cultured at 37 °C in a 5% CO₂ humidified atmosphere.

### Extraction and purification of COP-1

*COP* was extracted by a water extraction-alcohol precipitation method. COP was dissolved in distilled water and centrifuged at 4000 *rpm* for 10 min. Anhydrous ethanol was then added to the supernatant to achieve a final concentration of 80% [17]. Proteins were removed using the Sevag method. The resulting solution was subsequently applied to a Sephacryl S-200 HR column (2.6 × 140 cm, Whatman) and eluted with a 0.2 mol/L NaCl isocratic gradient at a constant flow rate of 0.6 mL/min. The main peak fraction was collected for dialysis and concentration. Further purification was performed on a Sephadex G-25 column (2.6 cm × 50 cm, Whatman) eluted with deionized water at a flow rate of 0.3 mL/min. The major peak was collected, dialyzed, lyophilized, and designated as COP-1.

### Sulfated modified polysaccharides

SCOP and SCOP-1 were obtained by the sulfur trioxide-pyridine method. Briefly, 0.2 g of COP and 0.2 g of COP-1 were each dissolved in 10 mL of anhydrous N, N-dimethylformamide (DMF). Subsequently, 1.2 g of sulfur trioxide-pyridine complex was added to each solution. The solution was sealed and stirred in an oil bath at 60 °C for 4 h. The mixture was cooled at room temperature in an ice water bath, pH adjusted to 7.0 ± 0.2 with 0.2 mol/L NaOH, 4 times the amount of ethanol was added, and the solution was alcohol-settled overnight, then centrifuged, and the supernatant was discarded, and the precipitate was placed in a 1500 Da dialysis bag, dialyzed, concentrated, and freeze-dried to obtain SCOP and SCOP-1. Barium chloride-gelatin turbidimetric method [10] was used to detect the degree of sulfate substitution in SCOP and SCOP-1. According to the absorbance value of the samples and the standard curve, DS was calculated according to the following formula:

$${\mathrm{DS}} = {1}.{\text{62S }}/ \, \left( {{32 } - { 1}.0{\mathrm{2S}}} \right) \, \times { 1}00\% ,{\text{ where S is the content of elemental sulfur in the samples}}{.}$$

### Homogeneity and molecular weight determination

The molecular weight curve was calibrated by dextran T series with different molecular weights of 1 k, 5 k, 12 k, 25 k and 50 k. Specific test conditions of HPGPC: Agilent High Performance Liquid System (1260 HPLC Pump, Inline Dgasser, 1260 Infinity Reflective Index Detector, Waters Ultra Hydrogel 250 (7.8 mm × 300 mm) column), Mobile phase: 0.2 mol/L sodium nitrate, flow rate: 0.6 mL/min, injection volume: 20 µL, column temperature: 30 ℃. COP (1 mg) was dissolved in distilled water, centrifuged at 12,000 *rpm* for 5 min, and then the supernatant was collected and analyzed by HPLC [23].

### Monosaccharide composition

According to the method for preparing a sample solution of the monosaccharide composition determination by Esposito et al. [9], the purified COP was prepared into a sample solution for later use.

Chromatographic analysis was performed on an Agilent 1260 HPLC system equipped with a 1260 HPLC pump, an in-line degasser, and an evaporative light-scattering detector (ELSD). Separation was achieved on an XBridge BEH Amide column (4.6 mm × 250 mm, 5 µm). The mobile phase consisted of (A) acetonitrile–water (80:20, v/v) containing 0.2% triethylamine and (B) acetonitrile–water (30:70, v/v) containing 0.2% triethylamine. Gradient elution was carried out as follows: 0–21.00 min, 100% A to 40% A; 21.00–33.00 min, 40% A to 100% A. The ELSD drift tube temperature was set at 30 ℃ and the nebulizer temperature at 30 ℃. The injection volume was 20 µL, the column temperature was maintained at 35 ℃, and the flow rate was 1.0 mL/min.

### NMR spectroscopy

COP-1 (100 mg) was dissolved separately in 99.9% DMSO‑d_6_ and 99.96% D₂O. The use of two different solvents allows observation of complementary structural features: D₂O provides optimal resolution for sugar ring protons, while DMSO‑d6 enables detection of hydroxyl protons through hydrogen bonding with the solvent. Gentle heating was applied if complete dissolution was not achieved. NMR spectra were recorded on an Agilent 400/54 MHz NMR spectrometer.

### Infrared (IR) spectroscopy

The KBr powder (400 mg) was separately mixed with dried COP-1 (2 mg) and SCOP-1 (2 mg), homogenized by grinding, and pressed into pellets. The samples were then further scanned using a Varian spectrometer (Varian 1000, Varian, USA) over the wavenumber range of 4000–500 cm^−1^.

### Antiviral activity assay

#### Cells and IAV (H1N1)

The cells were cultured in DMEM medium supplemented with 10% FBS and 1% pen-strep. They were maintained at 37 °C in a humidified 5% CO₂ atmosphere and split every 2–3 days. Using SPF-grade sterile chicken embryos, the influenza virus was amplified. All viral experiments were performed in Class II biological safety cabinet.

#### Cytotoxicity assay

The MTT assay was used to evaluate cell viability [40]. *C. pilosula* polysaccharide was two-fold serially diluted with DMEM to 20–1.25 mg/mL. Oseltamivir phosphate and DMEM served as positive and negative controls, respectively. MDCK/A549 cells were seeded in 96-well plates at 1 × 10^5^ cells/mL (100 µL/well) for 24 h, then treated with 100 µL of samples for 48 h. Subsequently, 10 µL of MTT (5 mg/mL) was added for 2–4 h. After removing the supernatant, 150 µL of DMSO was added, shaken for 10 min, and absorbance was measured at 490 nm.

#### Anti-influenza virus test

MDCK cells were seeded in 96-well plates and incubated for 24 h. Three treatment protocols were evaluated:

Prophylactic protocol: Cells were pretreated with test compounds for 24 h, then infected with 100 TCID₅₀ H1N1 virus for 2 h. The virus was removed, and virus maintenance medium was added for 48 h.

Therapeutic protocol: Cells were infected with 100 TCID₅₀ H1N1 virus for 2 h, then treated with test compounds for 48 h.

Direct virucidal protocol: Test compounds were mixed with 100 TCID₅₀ H1N1 virus (1:1, v/v) and incubated at 37 °C for 1 h. The mixture was added to cells for 2 h, then replaced with virus maintenance medium for 48 h.

Concentrations tested: COP at 0.625, 1.25, 2.5 mg/mL; SCOP and SCOP-1 at 4.88, 9.75, 19.5, 39, 78.1, 156.25 μg/mL. Based on cytotoxicity and preliminary screening, the following concentrations were selected for formal experiments: COP 1.25 mg/mL (direct virucidal); SCOP and SCOP-1 9.75 μg/mL (therapeutic and direct virucidal).

Four control groups were included: blank control, virus control, drug-treated control, and positive control (oseltamivir phosphate) [5,18]. After 48 h of incubation, cell viability was measured by MTT assay at 490 nm to calculate the viral inhibition rate.

#### Time-of-addition assay

MDCK cells were seeded in 6-well plates at a density of 2.5 × 10^4^ cells/well and incubated at 37 ℃ in a 5% CO_2_ atmosphere for 24 h. The medium was removed, and cells were infected with H1N1 at TCID_50_. COP (1.25 mg/mL), SCOP (9.75 µg/mL), or SCOP-1 (9.75 µg/mL) was added at different time points: 2 h before infection (− 2 h), at the time of infection (0 h), or 2, 4, 6, and 8 h post-infection. Virus control groups were set at each time point. At 12 h post-infection, cells were freeze-thawed three times at − 20 ℃, and viral titers in the supernatants were determined by TCID_50_ assay.

#### Virus-induced apoptosis assay

MDCK cells were seeded in 6-well plates at 2.5 × 10^4^ cells/well and cultured at 37 ℃ in 5% CO2 for 24 h. After treatment under direct virucidal modes and therapeutic modes, plates were placed on ice. The supernatant was collected, and cells were digested with EDTA-free trypsin for 1 min, then centrifuged at 300 g at 4 ℃ for 5 min. Cells were washed twice with cold PBS and resuspended in 100 µL 1 × Annexin V binding buffer. FITC-Annexin V (5 µL) and PI (5 µL) were added and incubated at room temperature in the dark for 10–15 min. Then 400 µL 1 × Annexin V binding buffer was added, and samples were analyzed by flow cytometry within 30 min.

#### qPCR analysis

A549 cells were cultured in 6-well plates and treated for 48 h. RNA was extracted by TRIzol method; reverse transcription and qRT-PCR were performed according to the Takara kit instructions. GAPDH was used as the internal reference gene for relative quantitative analysis, withprimer sequences as follows (Table 1).

**Table 1:** Primer sequences for qRT-PCR

Gene name		Primer sequence 5′-3′
IL-6	Forward	GACAGCCACTCACCTCTTCAGAAC
	Reverse	ATCGCCAAGCCTACCCACCTC
IL-8	Forward	AACTTTCAGAGACAGAGCACAC
	Reverse	CACACAGTGAGATGGTTCCTTCCG
MCP-1	Forward	TCAAAGAGAGGGTGGGTGTGTAGG
	Reverse	AGTCACCGTCTCTGGAAGCTAAGG
CCL-5	Forward	GGTCTCATCCCTCTTCTTCTCC
	Reverse	TTTCTTCTCTGGGTTGGCACACAC
TNF-α	Forward	GCTCCAGGCGGTGCTTGTTC
	Reverse	TTGGTTGGGAGAGTGGATGAAGG
TRAIL	Forward	ATAACCACAGCACCTCACGTCACC
	Reverse	CCAGTCACTCTCTCCACCCTCAC
GAPDH	Forward	AGTCAGCCGCATCTTCTTTT
	Reverse	CCCAATACGACCAAATCCGT

#### Western blot analysis

A549 cells were cultured in 6-well plates and treated for 48 h. Total cell protein was extracted using RIPA buffer, and the protein concentration was determined with the BCA protein assay kit. Then, protein sample (30 µg per lane) was separated by 10% SDS-PAGE and transferred onto PVDF membrane. The membrane was blocked with 2% blocking solution at room temperature for 2 h, followed by incubation with NF-κB p65 antibody overnight at 4 °C. After washing 3 times with TBST, the membrane was incubated with secondary antibody for 2 h at room temperature. Following final washes with TBST buffer, protein bands were detected and visualized using enhanced chemiluminescence reagent.

#### Statistical analysis

The experiment was repeated three times, and the data were statistically analyzed by SPSS29.0 (IBM USA) software. One-way ANOVA was used for comparison between groups, and the measurement data were expressed as mean ± standard deviation (X + SD). *P* < 0.05 was considered statistically significant.

## Results and discussion

### Extraction and purification

**Fig. 1:**
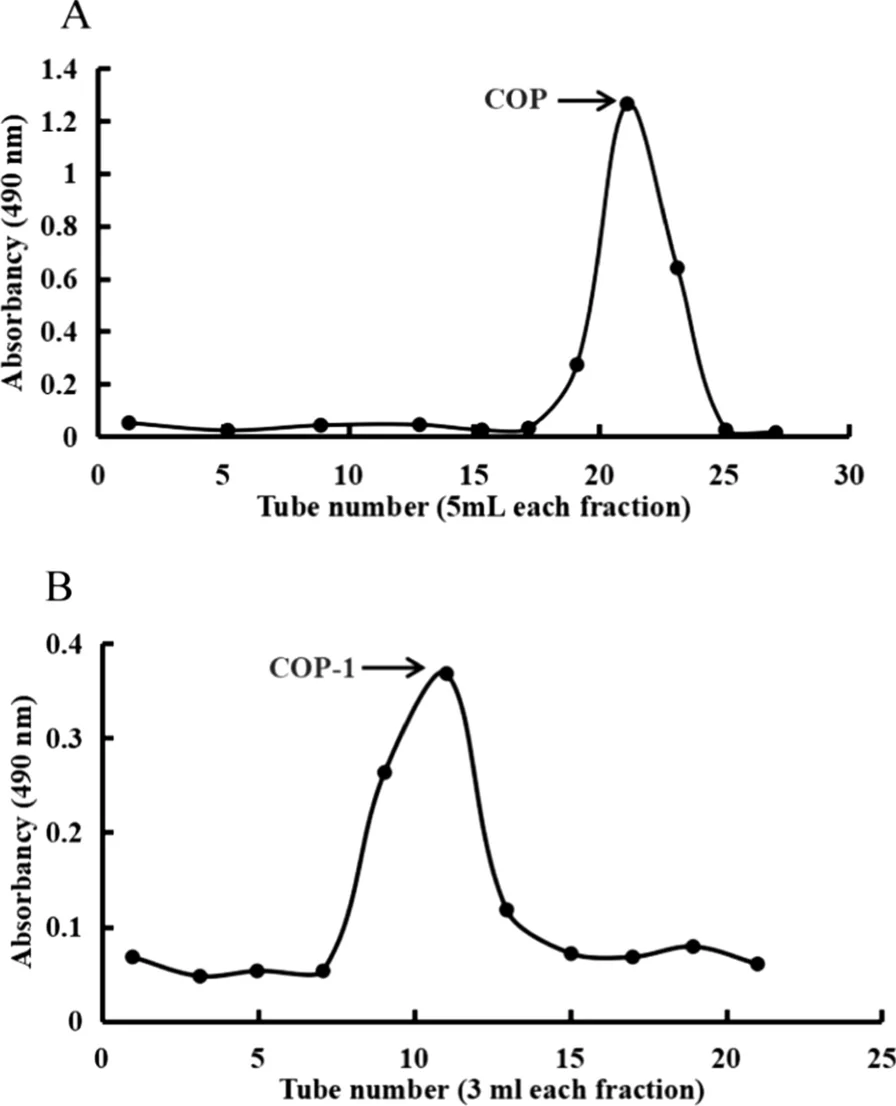
Elution profiles of polysaccharide by different packing material. **A** Elution profile of crude polysaccharide (COP) on a Sephacryl S-200 HR column (2.6 × 140 cm), eluted with 0.2 M NaCl at 0.6 mL/min. **B** Further purification of COP-1 on a Sephadex G-25 column (2.6 × 50 cm), eluted with deionized water at 0.3 mL/min. Fractions were monitored by the phenol–sulfuric acid method at 490 nm

The Sephacryl S-200HR column chromatography of COP yielded a single major peak (Fig. 1A). After further purification on a Sephadex G-25 column, COP-1 also exhibited a single symmetrical peak (Fig. 1B), indicating that COP-1 is a homogeneous polysaccharide.

### Sulfate content of SCOP and SCOP-1

**Fig. 2:**
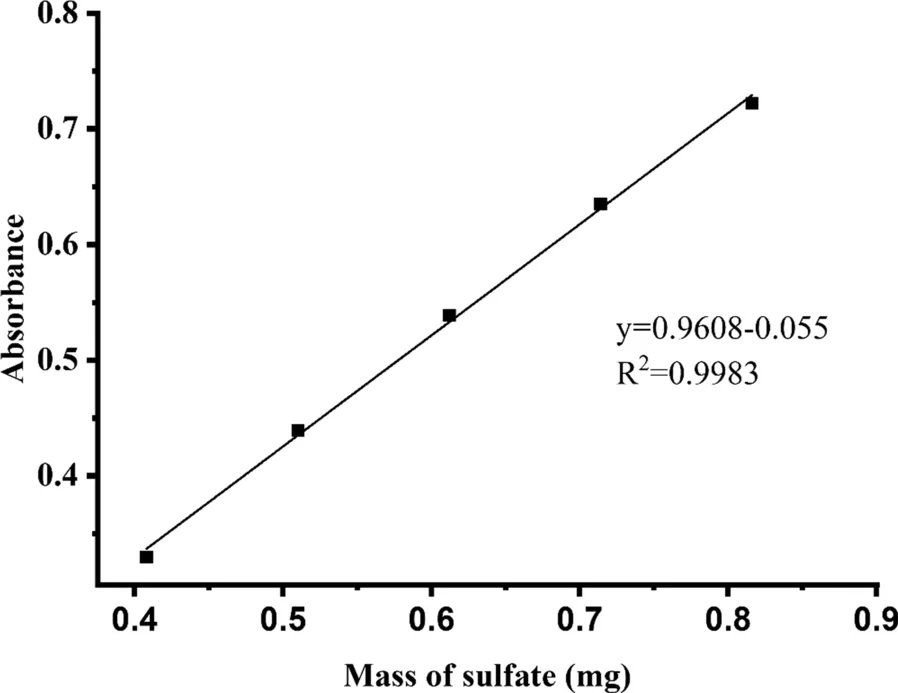
Standard curve for sulfate content determination. Various concentrations of Na_2_SO_4_ (0.408–0.816 mg) were analyzed by the barium chloride–gelatin turbidimetric method at 360 nm. The regression equation was Y = 0.9608X − 0.055 (R^2^ = 0.9983)

The sulfate standard curve was established as follows (Fig. 2): Sample absorbance values were substituted into the regression equation (Y = 0.9608X − 0.055, R2 = 0.9983). The sulfate substitution degrees (DS) of SCOP and SCOP-1 were calculated as 1.792 and 1.403, respectively.

### Homogeneity and molecular weight determination

**Fig. 3:**
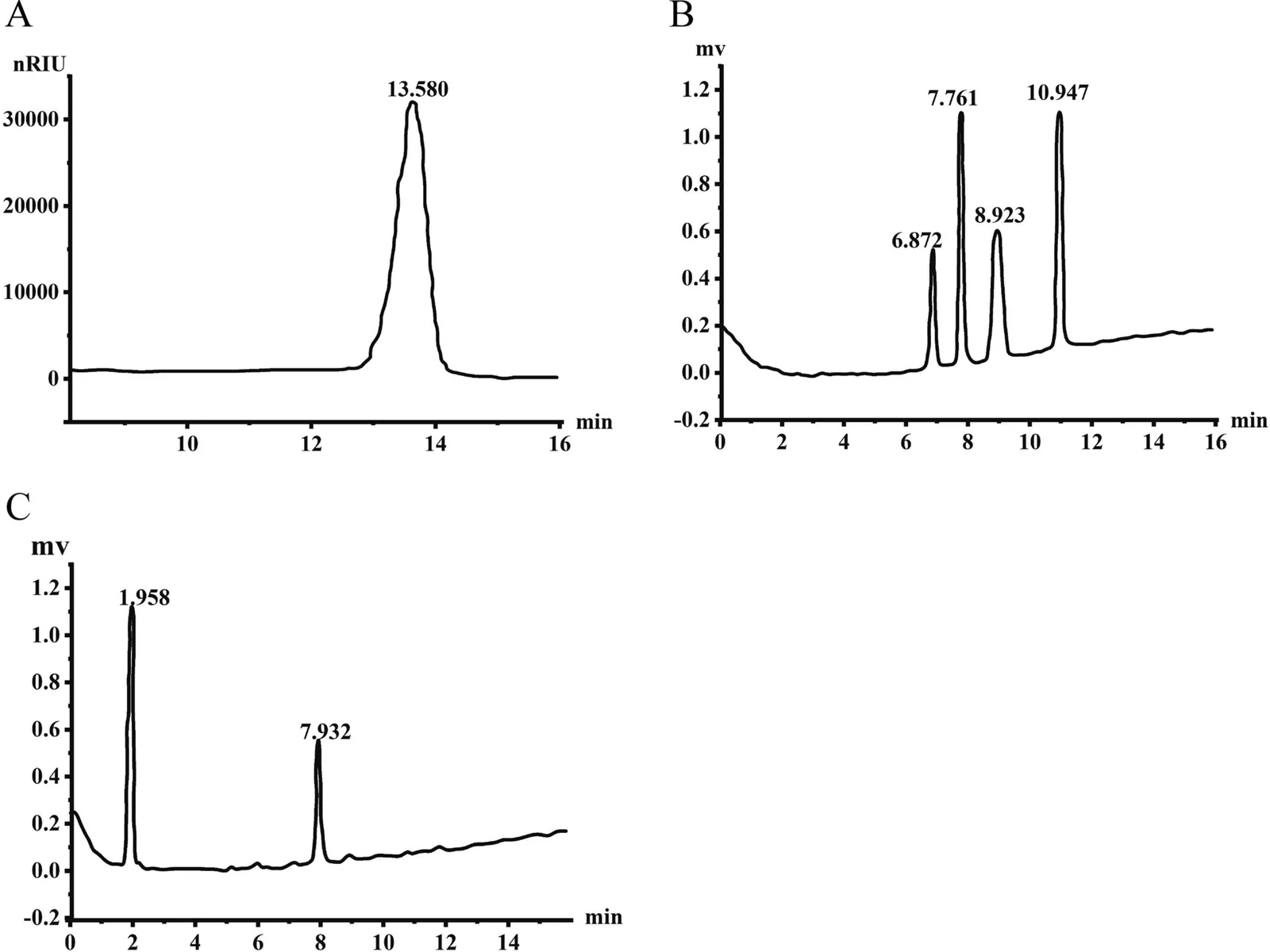
**A** HPGPC profile of polysaccharide COP-1 (retention time of COP-1: t_R_ = 13.580 min); **B** HPLC chromatograms of four standard monosaccharides. Retention time of four kinds of monosaccharide: Xylose t_R_ = 6.872 min; Fructose t_R_ = 7.761 min; Mannose t_R_ = 8.923 min; Glu- cose t_R_ = 10.947 min; **C** Hydrolyzing of COP-1 (fructose t_R_ = 7.932 min). The peak at approximately 1.9 min is the dead time peak (solvent front), not a monosaccharide. (Reproduced with permission from Liu et al. [25]. Copyright © 2016 Genomics and Applied Biology.)

COP-1 exhibited a symmetric single peak in HPGPC gel filtration chromatography (Fig. 3A), indicating that it is a homogeneous polysaccharide, whose structural analysis was first reported by Liu et al. [25]. According to the standard curve of molecular weight: $\mathrm{log}M=2.544+0.6998{\rm X}-0.0473{{\rm X}}^{2}$, the average molecular weight of the homogeneous polysaccharide (COP-1) was calculated to be 2.1 kDa.

### Monosaccharide composition

Monosaccharide composition of COP-1 was determined by direct hydrolysis of polysaccharide samples, followed by HPLC analysis. As shown in the previous structural analysis [25], the HPLC chromatograms of monosaccharide and polysaccharide hydrolysis are presented in Fig. 3B and C. The early eluting peak at approximately 1.9 min in Fig. 3C corresponds to the dead time (t_0_), representing unretained solvent components rather than monosaccharides. Compared with monosaccharides, the peak time of polysaccharide hydrolysate showed that COP-1 was mainly composed of fructose.

### NMR spectroscopy

The nuclear magnetic resonance (NMR)-based structural analysis of *Codonopsis pilosula* polysaccharide was described in our earlier work [25]. NMR spectra were recorded in both D_2_O and DMSO-*d*_6_ to obtain complementary structural information. In D_2_O, rapid proton exchange with the solvent obscures hydroxyl signals but provides excellent resolution for sugar ring protons. In DMSO-*d*_6_, hydrogen bonding between the solvent and sugar hydroxyl groups stabilizes the OH protons, allowing their detection.

**Fig. 4:**
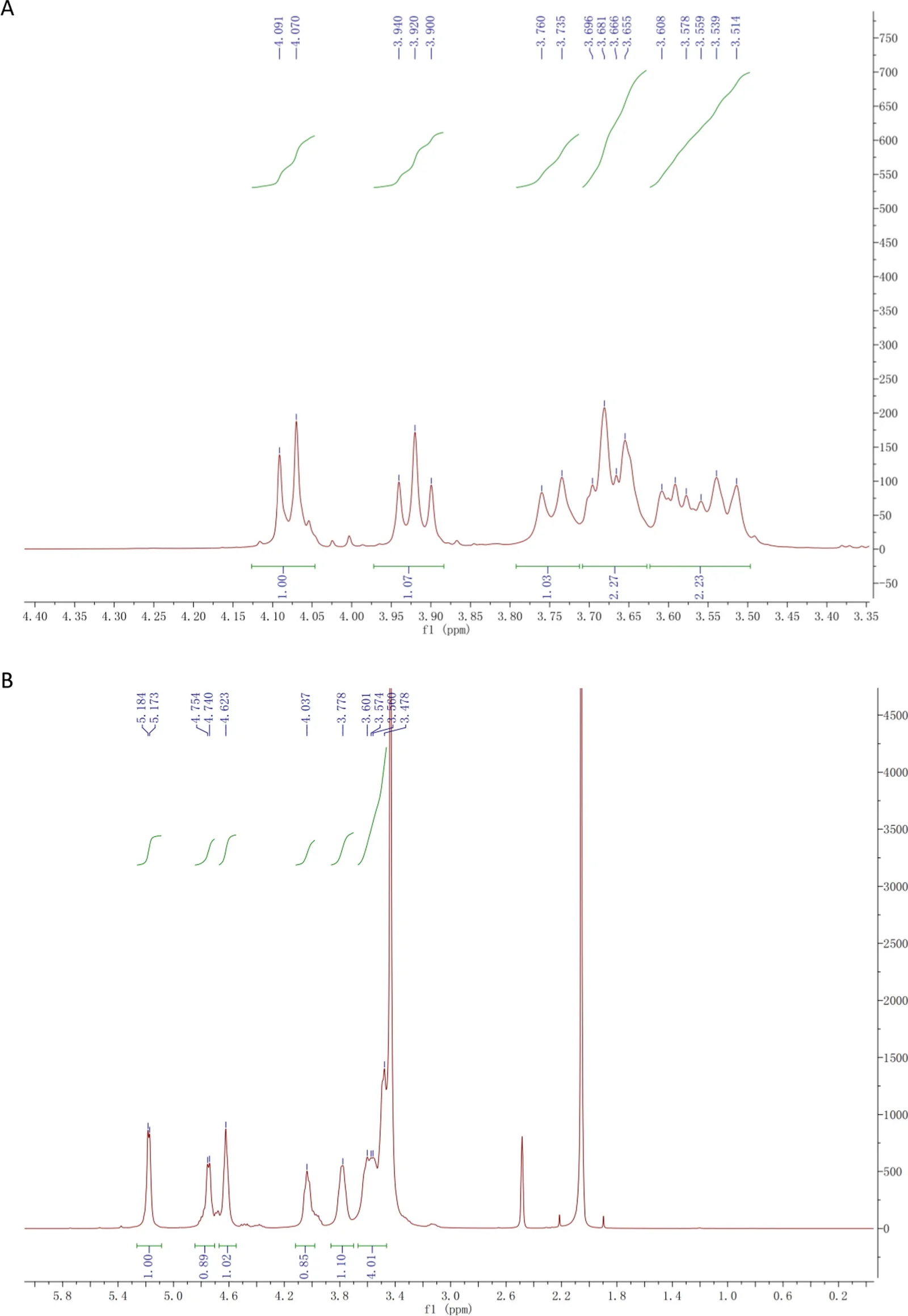

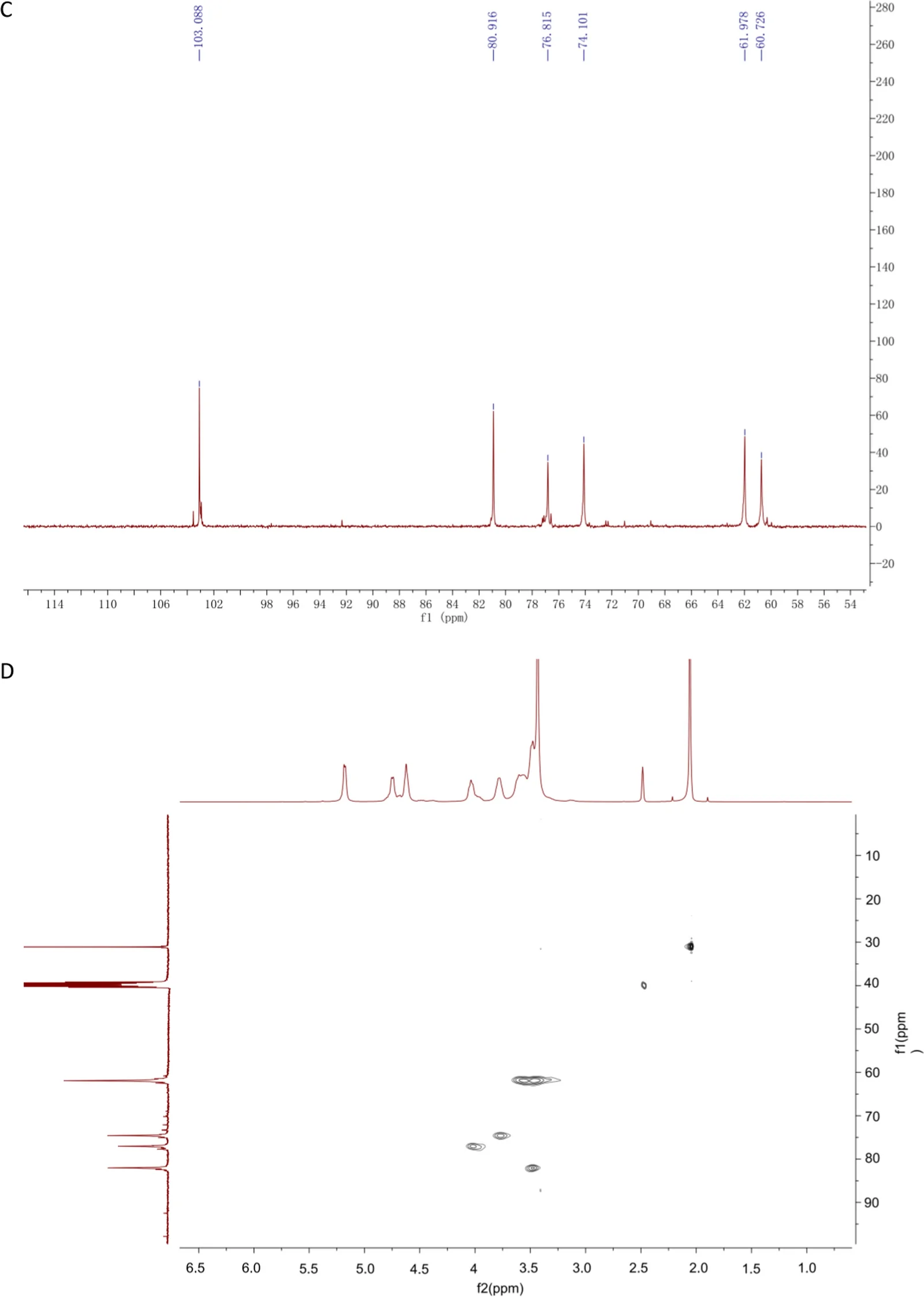

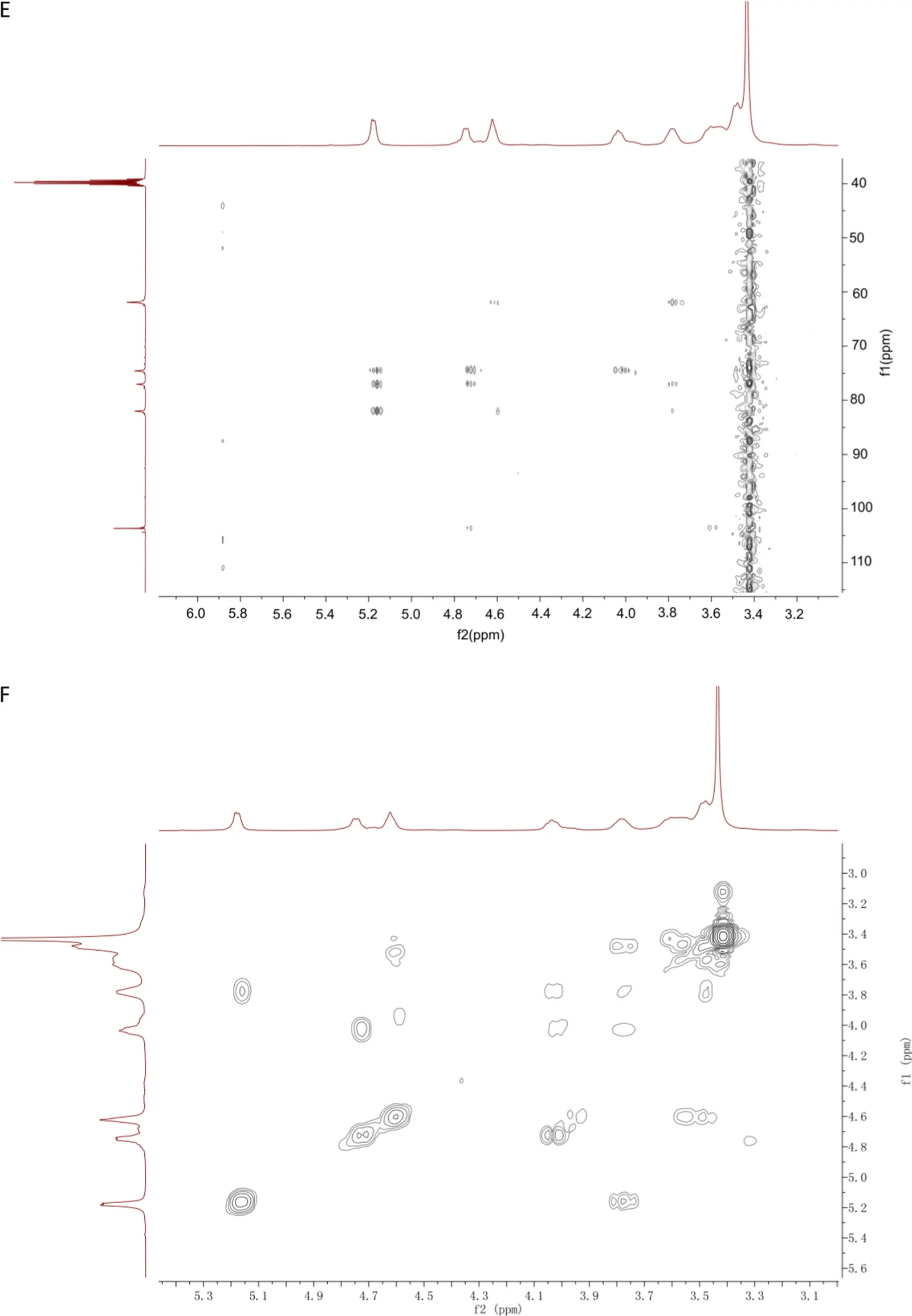

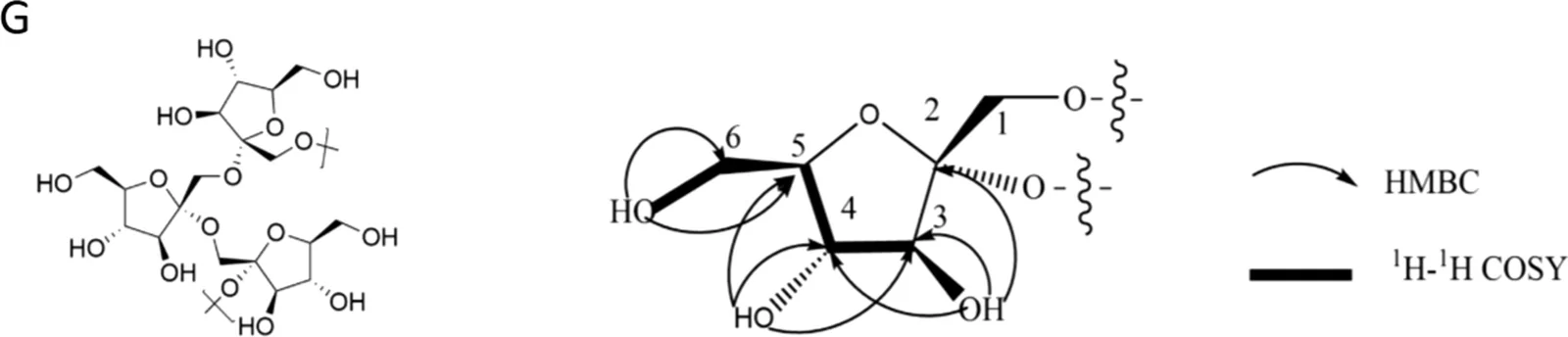
NMR Spectrum of COP-1. **A** 1H NMR (D_2_O); **B** 1H NMR (DMSO-*d*_6_); **C** 13C NMR (D_2_O); **D** HSQC (DMSO-*d*_6_); **E** HMBC (DMSO-*d*_6_); **F** 1H-1H COSY (DMSO-*d*_6_); **G** Key HMBC correlations of fructose unit. (Adapted with permission from Liu et al. [25]. Copyright © 2016 Genomics and Applied Biology)

The ^1^H NMR spectrum of COP-1 (Fig. 4A, in D_2_O) revealed seven downfield proton signals: *δ*_H_ 4.09 (1H, d, *J* = 8.4 Hz, H-3), 3.94 (1H, t, *J* = 8.0 Hz, H-4), 3.76 (1H, d, *J* = 10.0 Hz, H-1a), 3.69 (2H, m, H-5, H-6a), and 3.60 (2H, m, H-6b, H-1b). When measured in DMSO-*d*_6_ (Fig. 4B), three hydroxyl proton signals were observed at *δ*_H_ 5.18 (1H, d, *J* = 4.4 Hz, 4-OH), 4.75 (1H, d, *J* = 5.6 Hz, 3-OH), and 4.62 (1H, s, 6-OH). The 13C NMR, DEPT (Fig. 4C, in D₂O), and HSQC (Fig. 4D, in DMSO-*d6*) spectra displayed six carbon signals: C-1 (*δ* 62.0), C-2 (*δ* 103.1), C-3 (*δ* 76.8), C-4 (*δ* 74.1), C-5 (*δ* 80.9), and C-6 (*δ* 60.7). Comparative analysis with literature [45] suggested a fructofuranose unit. The anomeric carbon signal at *δ*_C_ 103.1 indicated a β-configuration of the sugar ring [42]. The HMBC spectrum (Fig. 4E, in DMSO-*d*₆) revealed long-range correlations between hydroxyl protons and carbon atoms (e.g., 3-OH/C-2, C-3, C-4; 4-OH/C-3, C-4, C-5; 6-OH/C-5, C-6), as illustrated in Fig. 4G. Additionally, the ^1^H–^1^H COSY spectrum (Fig. 4F, recorded in DMSO‑d₆) revealed clear correlations between each hydroxyl proton and its adjacent methine/methylene proton, specifically 6-OH (*δ*_H_ 4.62, s)/H-6 (*δ*_H_ 3.69, m), 3-OH (*δ*_H_ 4.75, d, *J* = 5.6 Hz)/H-3 (*δ*_H_ 4.09, d, *J* = 8.4 Hz), and 4-OH (*δ*_H_ 5.18, d, *J* = 4.4 Hz)/H-4 (*δ*_H_ 3.94, t, *J* = 8.0 Hz). In addition, the spectrum established the complete coupling network of the sugar ring protons, with sequential correlations observed for H‑6/H‑5, H‑5/H‑4, and H‑4/H‑3. Taken together, these correlation signals provide unambiguous evidence confirming that the molecular structure is consistent with the connectivity and stereochemistry depicted in Fig. 4G.

These correlation patterns indicate that the hydroxyl groups at OH-1 and OH-2 were involved in glycosidic bond formation. Finally, the structure of polysaccharide COP-1 can be determined as follows: [→ 1)-β-D-fru-(2 → 1)-] n. Detailed 1H and 13C NMR spectroscopic data for COP-1, including chemical shifts, multiplicities, coupling constants, and solvent-specific assignments in both D_2_O and DMSO-*d*_6_, are summarized in Table S1. Key HMBC and COSY correlations confirming the glycosidic linkages and hydroxyl group positions are listed in Table S2.

### IR spectroscopy

**Fig. 5:**
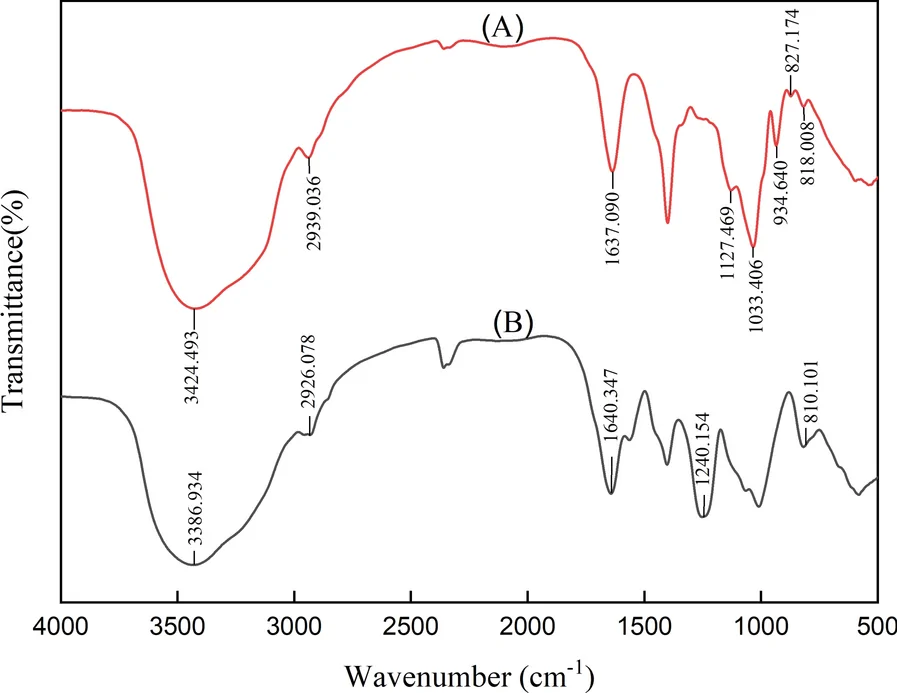
FT-IR spectra of COP-1 (**A**) and SCOP-1 (**B**). Spectra were recorded on a Varian 1000 spectrometer using KBr pellets (400 mg KBr + 2 mg sample) over 4000–500 cm^−1^

The infrared spectra of COP-1 polysaccharides reflect their functional groups and chemical bonds (Fig. 5). The response of the absorption peak 3200–3500 cm^−1^ is caused by the tensile vibration of –OH. The absorption at 2939 cm^−1^ is due to the stretching vibration of –CH [4], while the signal at 1637 cm^−1^ corresponds to water [19]. The signals absorbed at nearby 1127 cm^−1^ and 1033 cm^−1^ indicate the presence of a furan ring configuration [3,47], which also indicates that COP-1 is a furan glycosidic bond. The polysaccharide configuration was deduced to be β-D-furan fructose from the absorption at 934 cm^−1^, 818 cm^−1^ [22,48]. There was no absorption at 1740 cm^−1^, suggesting that there is no uronic acid in the COP-1 structure [6].

The FTIR spectrum of SCOP-1 is shown in Fig. 5. The absorption peaks at 3386 cm^−1^, 2926 cm^−1^, and 1640 cm^−1^ correspond to the stretching vibration of –OH bonds in hydroxyl groups of polysaccharides, the stretching vibration of –CH bonds (e.g., –CH_2_), and the characteristic absorption of bound water in polysaccharide molecules, respectively. Additionally, the regions of 1400–1200 cm^−1^ and 1200–1100 cm^−1^ exhibit typical polysaccharide absorption bands. Besides retaining the characteristic peaks of polysaccharides, SCOP-1 also displays distinct absorption bands attributed to sulfate groups: a peak at 1240 cm^−1^, assigned to the asymmetric stretching vibration of S=O, and a peak near 810 cm^−1^, corresponding to the C–O–S stretching vibration. These results confirm the successful sulfation modification of codonopsis polysaccharide [2,13,15].

### Antivirus activity in vitro

#### Cultivation of influenza virus H1N1 and determination of TCID_50_

The titer of the influenza H1N1 virus reaches up to 1:1280 after proliferation in the allantoic cavity of chicken embryos [44]. Therefore, a virus solution with a titer of 1:1280 was used to determine the TCID_50_. According to the Reed-Muench formula, the TCID_50_ of the virus is 10^–6.5^ per 100 µL.

#### Detection of drug cytotoxicity to MDCK cells

**Fig. 6:**
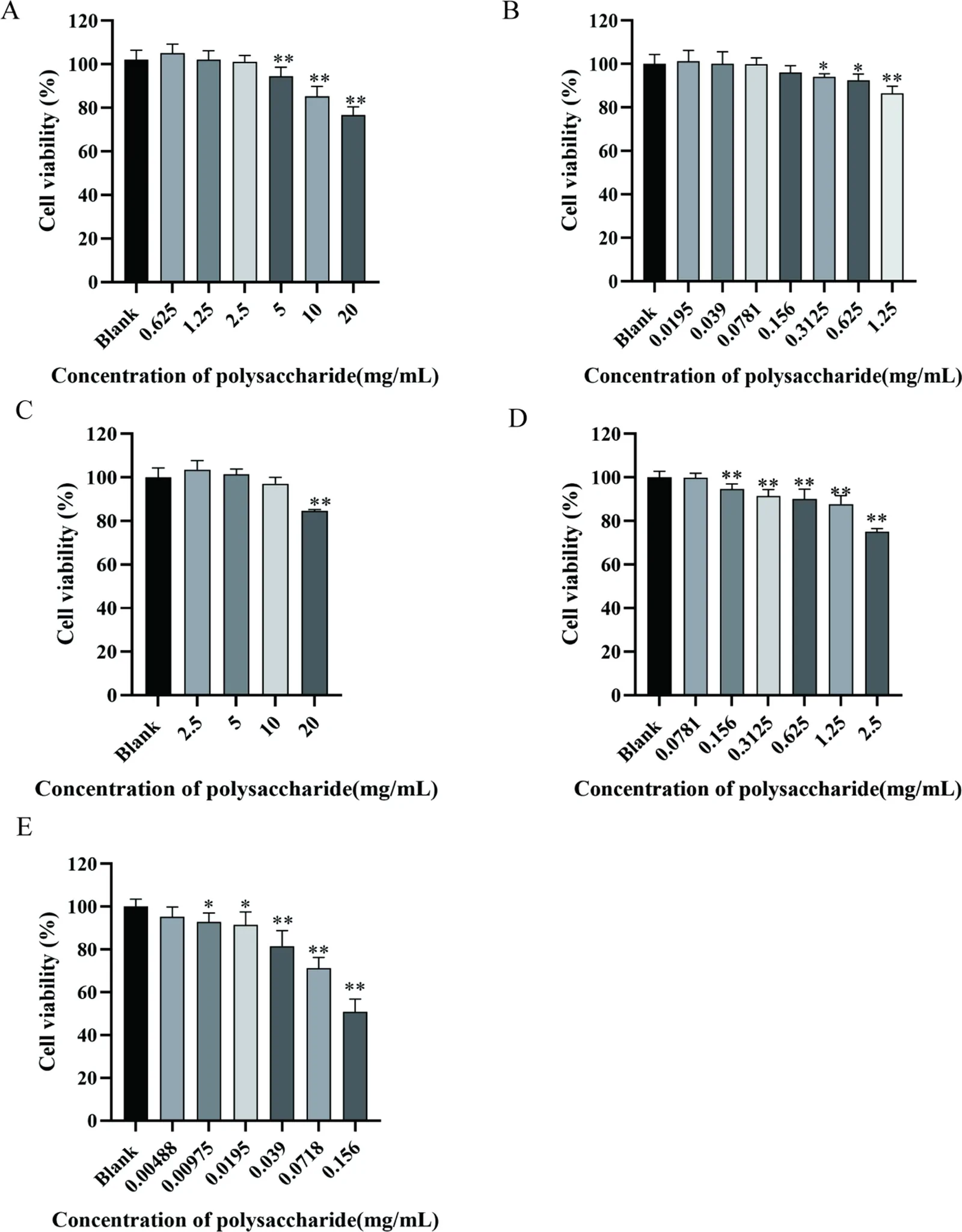
Cytotoxicity of compounds on MDCK cells. Cells were treated with serial dilutions of COP (**A**), SCOP (**B**), COP-1 (**C**), SCOP-1 (**D**), or oseltamivir (**E**) for 48 h. Cell viability was determined by MTT assay at 490 nm. Data are presented as mean ± SD (n = 6). **P < 0.01, *P < 0.05 vs. control group

The cytotoxicity of test compounds on MDCK cells was evaluated using MTT assay [20], and over a certain range, the mortality of MDCK cells was positively correlated with the amount of the compounds. The maximum non-toxic concentration of the compounds for MDCK cells (vs. control, *P* > 0.05) was selected as the cell stimulation concentration for subsequent experiments. As shown in Fig. 6, the maximum non-toxic concentrations of COP, COP-1, SCOP, SCOP-1, and Oseltamivir were 2.5 mg/mL, 10 mg/mL, 156.25 µg/mL, 78.1 µg/mL, and 4.88 µg/mL, respectively. Since COP is almost non-toxic in the range of 0–20 mg/mL, the maximum non-toxic concentration was selected for subsequent experiments.

#### Determination of drug action against influenza viruses

**Fig. 7:**
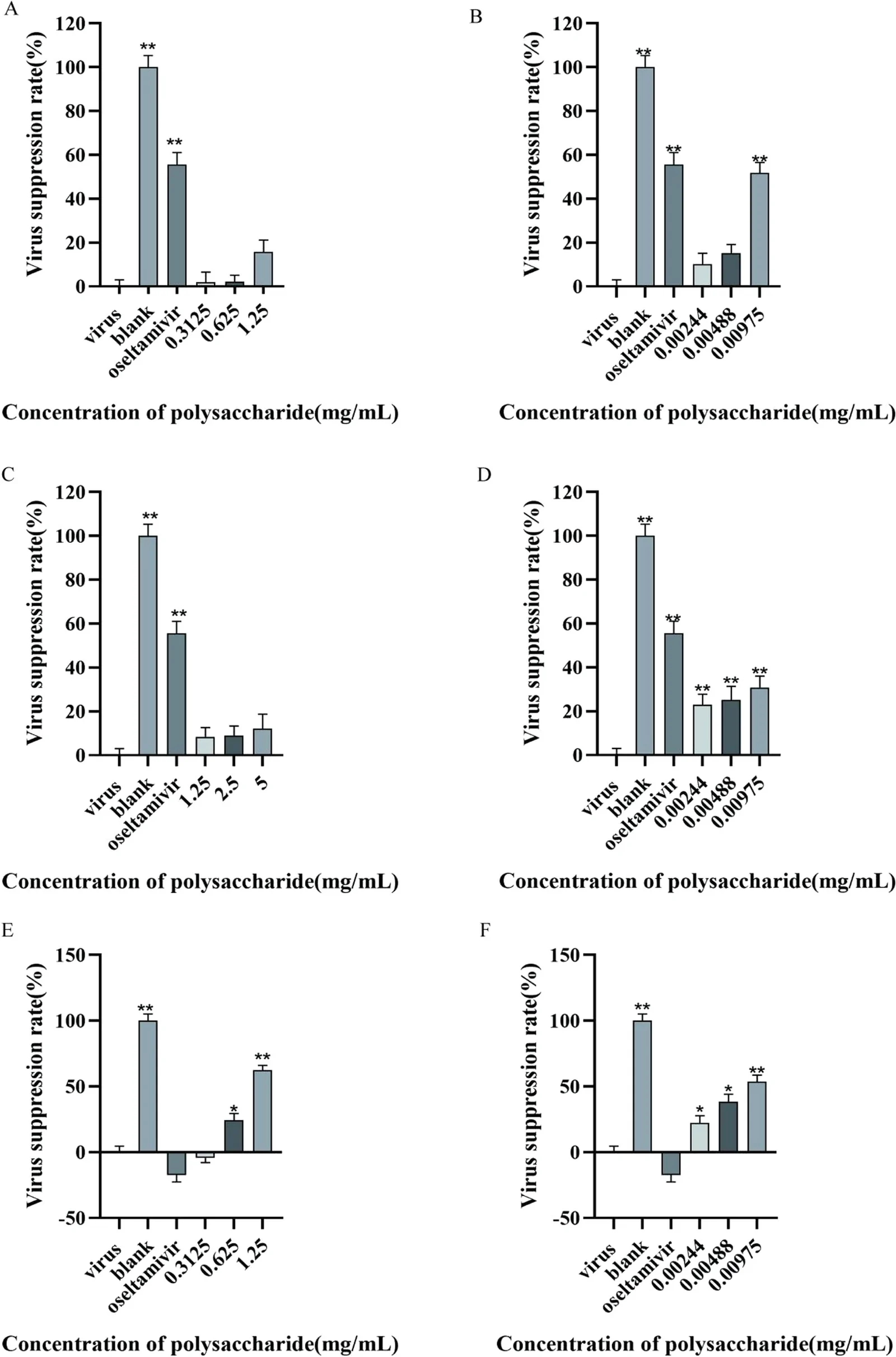

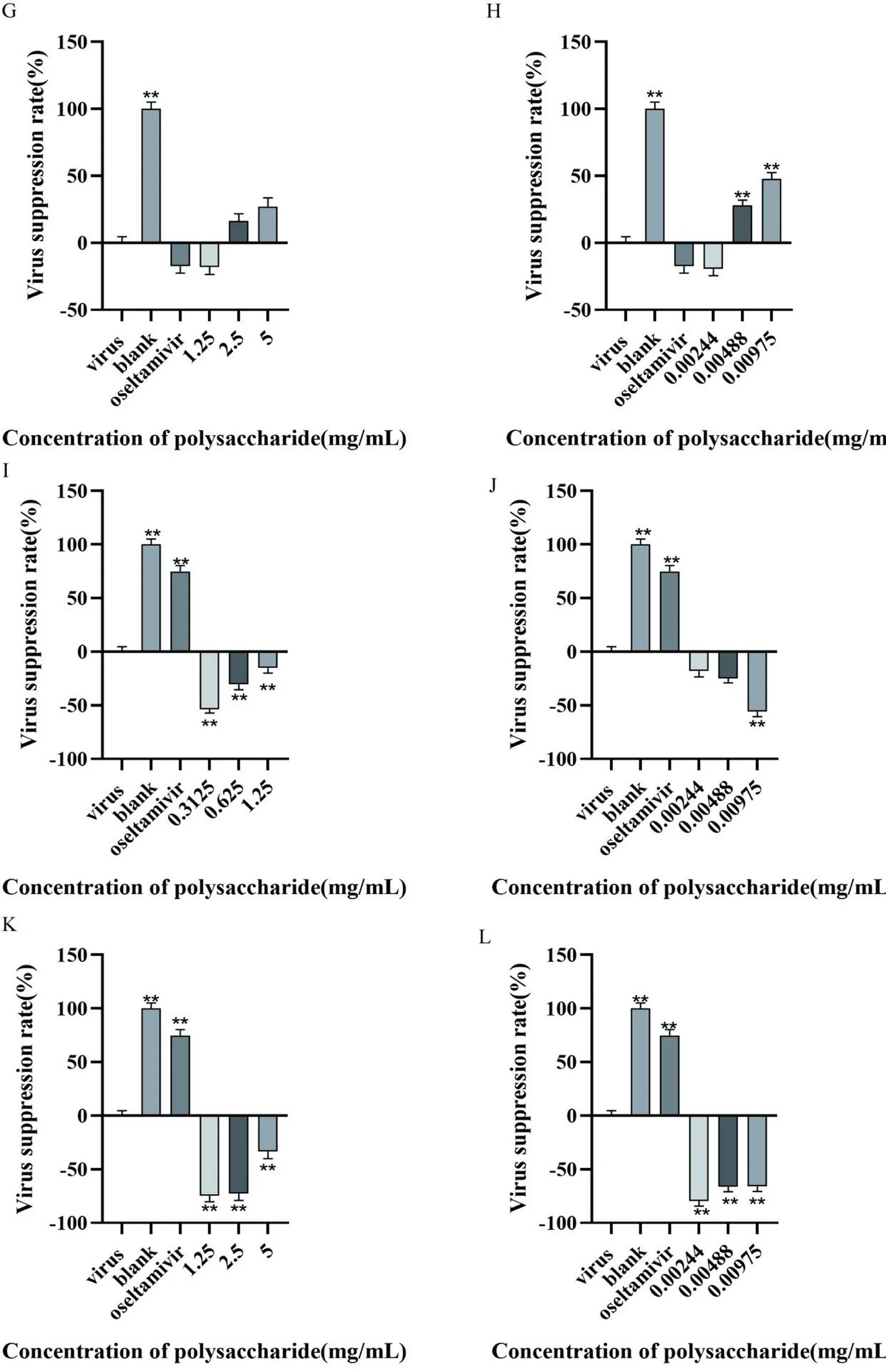
Antiviral activity of various polysaccharides against IAV (H1N1,100 TCID_50_). Viral inhibition was determined by MTT assay at 490 nm. **A**–**D** Therapeutic mode: **A** COP (1.25 mg/mL), **B** SCOP (9.75 µg/mL), **C** COP-1 (10 mg/mL), **D** SCOP-1 (9.75 µg/mL). **E**–**H** Direct virucidal mode: **E** COP (1.25 mg/mL), **F** SCOP (9.75 µg/mL), **G** COP-1 (10 mg/mL), **H** SCOP-1 (9.75 µg/mL). **I**–**L** Prophylactic mode: **I** COP (2.5 mg/mL), **J** SCOP (156.25 µg/mL), **K** COP-1 (10 mg/mL), **L** SCOP-1 (78.1 µg/mL). Data are presented as mean ± SD (n = 6). **P < 0.01, *P < 0.05 vs. virus group

Oseltamivir phosphate (4.88 µg/mL) was used as positive control. Under the therapeutic mode, COP, SCOP, and SCOP-1 exhibited inhibitory effects against H1N1. Specifically, at a concentration of 9.75 µg/mL, SCOP and SCOP-1 achieved inhibition rates of 51.7% and 30.88%, respectively, whereas COP-1 showed no inhibitory activity (Fig. 7A–D). Under the direct virucidal mode, COP demonstrated a direct virucidal effect against H1N1 at a concentration of 1.25 mg/mL, achieving an inhibition rate of 62.42% (Fig. 7E). And at a concentration of 9.75 µg/mL, SCOP and SCOP-1 attained inhibition rate of 53.74% and 47.51%, respectively. Notably, no inhibitory effect was observed under the prophylactic mode (Fig. 7I–L). Compared to COP and COP-1, SCOP and SCOP-1 showed superior inhibitory activity against H1N1. These results indicate that sulfation modification significantly enhances the anti-H1N1 activity of the polysaccharides.

The biological activity of polysaccharides is influenced by their molecular structure; chemical modifications can alter spatial conformation, molecular weight, and the type, number, and position of substituents, thereby enhancing or conferring new activities [33]. COP-1 is a homogeneous fructan (2.1 kDa) with low molecular weight and no charged groups, which may limit its effective interaction with viral particles or host cells. After sulfation, the introduction of negatively charged sulfate groups significantly enhanced the activity of SCOP and SCOP-1. This is consistent with previous reports: lentinan itself has no anti-HIV activity, but after sulfation, it effectively prevents HIV-induced cytopathic effects [1]. Numerous studies have shown that sulfated polysaccharides inhibit influenza virus, HIV, HSV and other viruses [11,31,33] and our results are consistent with these findings.

#### Effects of time-of-addition on viral replication

**Fig. 8:**
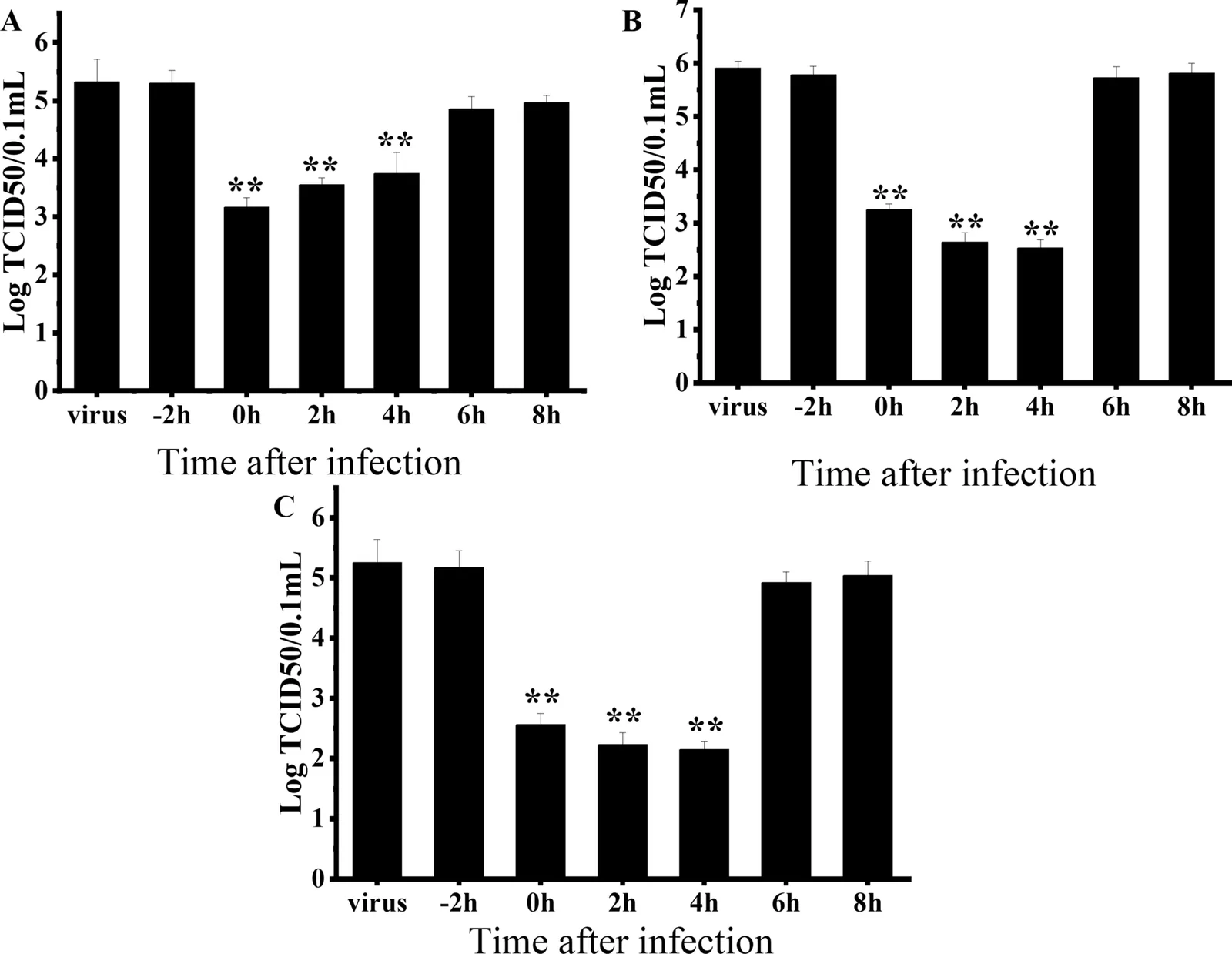
Time-of-addition assay. MDCK cells were infected with H1N1 (100 TCID₅₀). Compounds were added at indicated times post-infection. Viral titers were measured at 12 hpi by TCID₅₀. **A** SCOP-1; **B** COP; **C** SCOP. Compounds were added at the following concentrations: COP (1.25 mg/mL), SCOP (9.75 µg/mL), and SCOP-1 (9.75 µg/mL). Data are presented as mean ± SD (n = 3). **P < 0.01, vs. virus group

Viral yields were significantly reduced when COP, SCOP, or SCOP-1 were added at 0–4 h post-infection (Fig. 8). In contrast, treatment at − 2 h, 6 h, or 8 h showed no significant effect on viral titers. These results indicate that COP, SCOP, and SCOP-1 inhibit influenza virus replication primarily during the early stage of the viral replication cycle.

The antiviral activity of sulfated polysaccharides is generally attributed to electrostatic interactions between the negatively charged sulfate groups and positively charged regions on viral surface proteins, thereby blocking viral adsorption or entry into host cells [24]. The specific molecular target requires further investigation.

#### Effects on virus-induced apoptosis

**Fig. 9:**
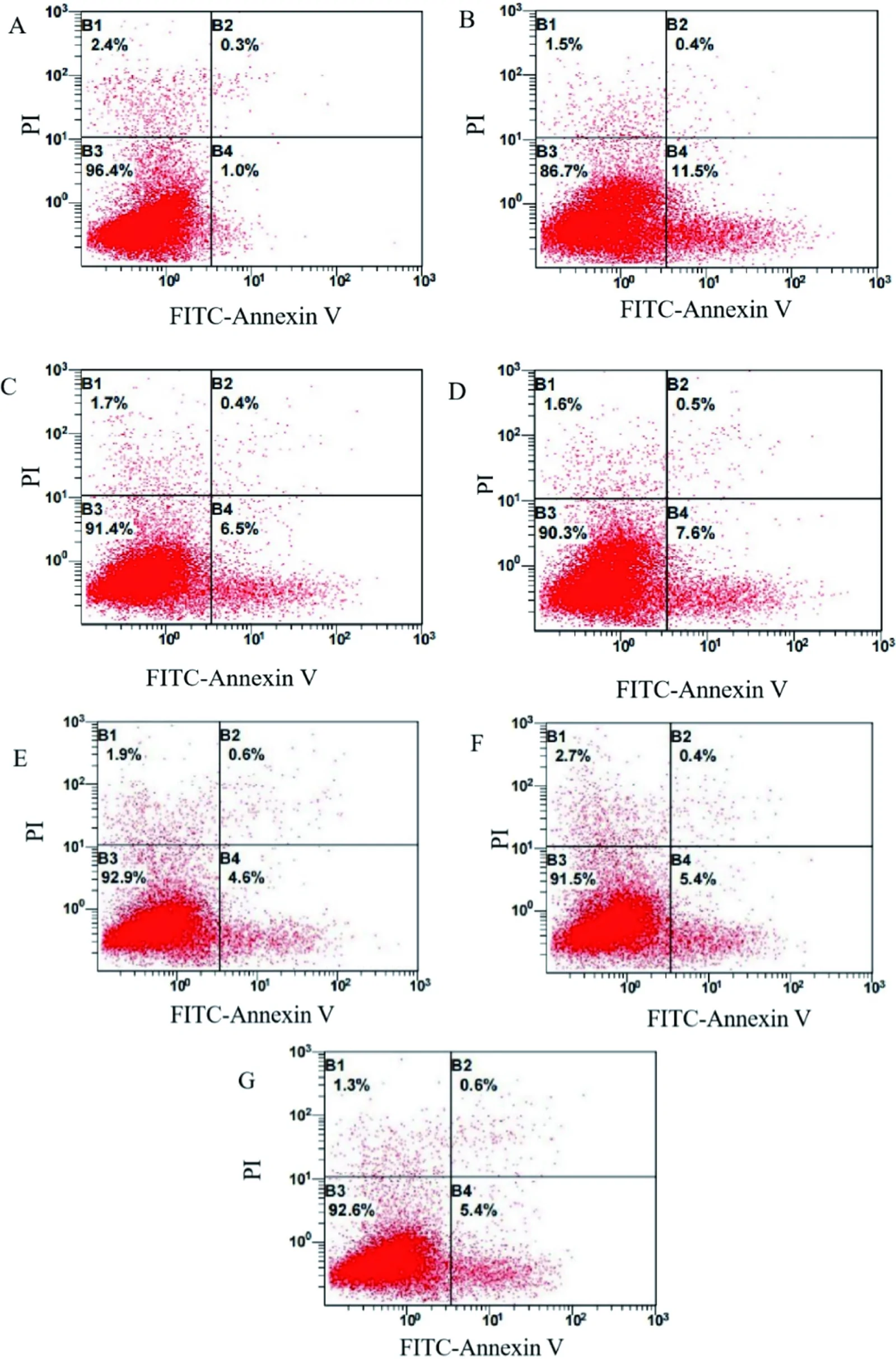
Inhibitory effect of compounds on H1N1-induced apoptosis in MDCK cells. Cells were infected with H1N1 (100 TCID_50_). **A** Normal control; **B** Virus control; **C** SCOP (therapeutic mode, 9.75 µg/mL); **D** SCOP-1 (therapeutic mode, 9.75 µg/mL); **E** COP (direct virucidal mode, 1.25 mg/mL); **F** SCOP (direct virucidal mode, 9.75 µg/mL); **G** SCOP-1 (direct virucidal mode, 9.75 µg/mL). Apoptosis was assessed by Annexin V-PI staining and flow cytometry. Data are representative of three independent experiments

The impact of COP and its sulfated derivatives on H1N1-induced apoptosis in host cells was assessed. As shown in Fig. 9, the apoptotic rates in the H1N1-infected control group were 11.9% (B), markedly higher than the 1.3% observed in the normal control group (A). This effect was mitigated by all treatments, with apoptotic rates significantly reduced to 6.9% (SCOP therapeutic mode), 8.1% (SCOP-1 therapeutic mode), 5.2% (COP direct virucidal mode), 5.8% (SCOP direct virucidal mode), and 6.0% (SCOP-1 direct virucidal mode). The results demonstrate that COP, SCOP, and SCOP-1 inhibit influenza virus-induced apoptosis in vitro, primarily during the early phase of apoptosis, aligning with their previously observed mechanism of action in the early stage of viral replication. Influenza virus exploits host cell apoptosis to facilitate progeny virus release and spread; thus, inhibition of apoptosis can effectively block viral dissemination. All three compounds inhibited apoptosis. The NF-κB pathway plays a dual role in the regulation of apoptosis, and the inhibition of NF-κB by these compounds [14] may contribute to their anti-apoptotic effects.

#### Effects of compounds on H1N1-induced cytokine mRNA expression under different administration modes

Influenza A virus invades the host through airborne transmission, triggering both innate and adaptive immune responses. The immune activation often leads to the excessive production of cytokines within a short period, a phenomenon known as a “cytokine storm” [14]. The host defense against influenza viruses is largely dependent on immune regulation. Viral infection induces an inflammatory response, prompting various immune cells to release high concentrations of pro-inflammatory factors, including IL-6, IL-8, TNF-*α*, CCL-5, MCP-1, and TRAIL [16]. Consequently, controlling the overexpression of these pro-inflammatory cytokines and chemokines to alleviate the cytokine storm has become a critical focus in anti-influenza research [46].

**Fig. 10:**
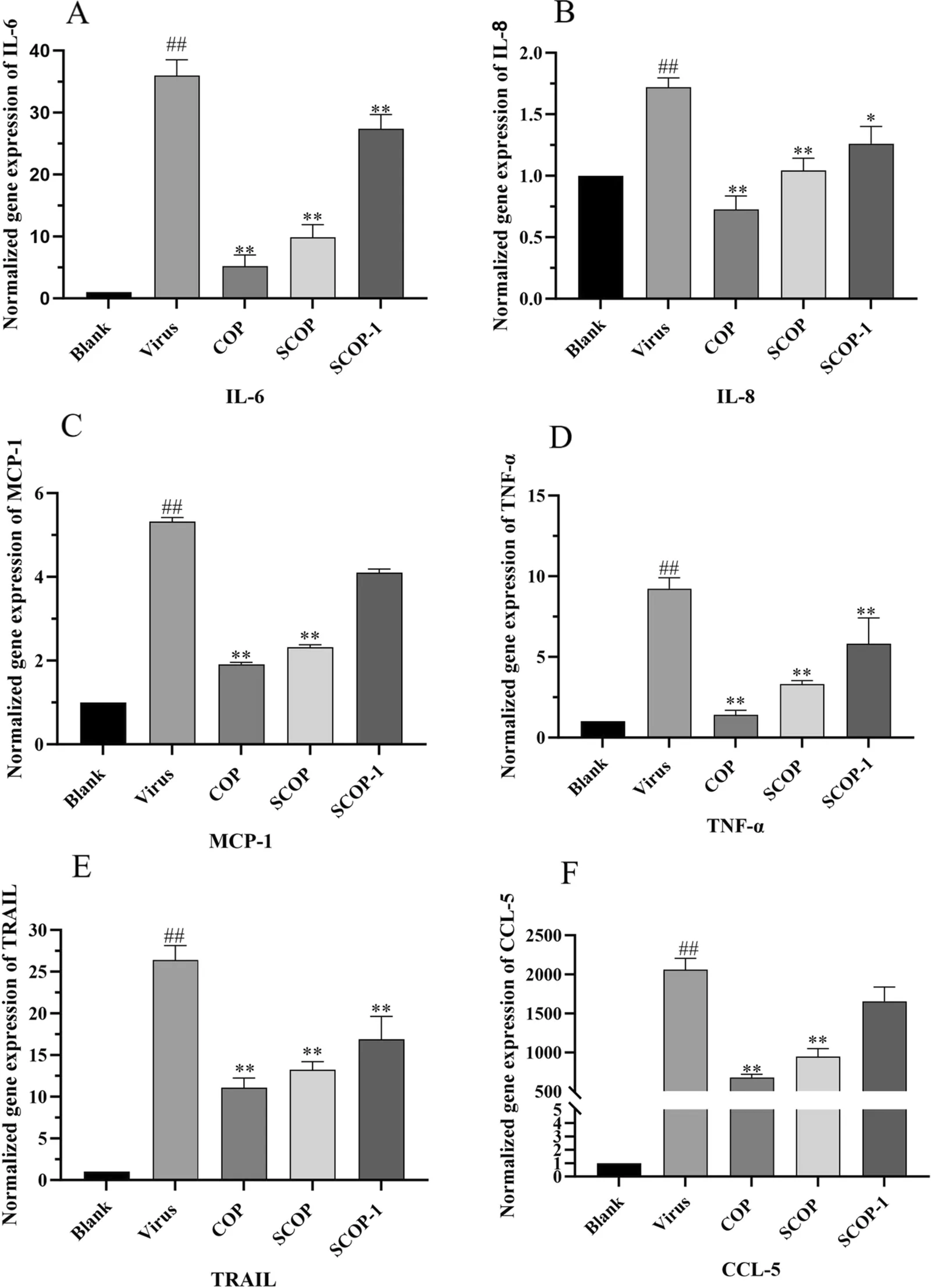

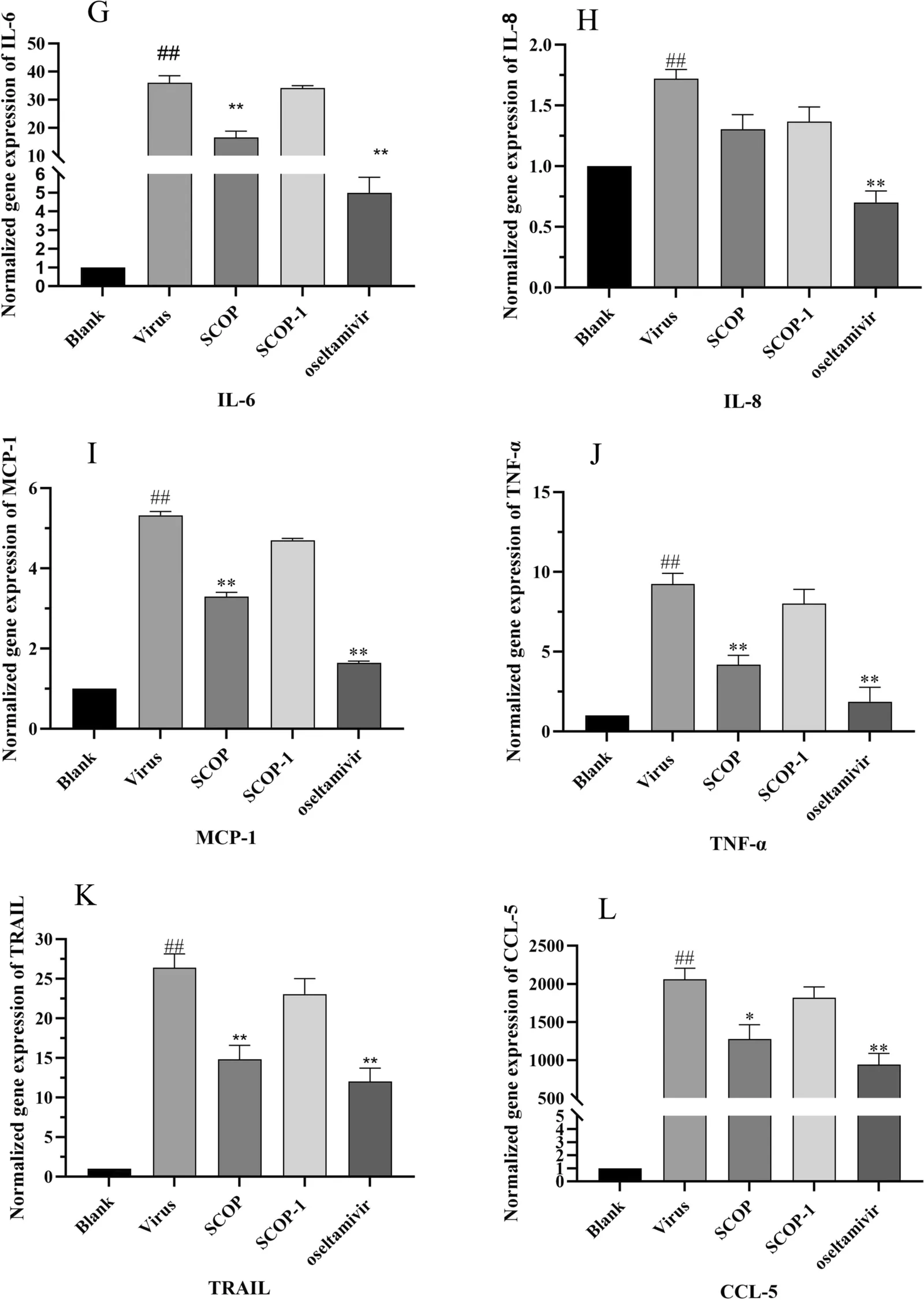
Effect of compounds on H1N1-induced cytokine mRNA expression in A549 cells. Cells were infected with H1N1 for 48 h. Total RNA was extracted, and the mRNA levels of *IL-6, IL-8, MCP-1, TNF-α, TRAIL* and *CCL-5* were quantified by qRT-PCR using the 2^−△△Ct^ method, with GAPDH as the internal control. Direct virucidal mode (**A**–**F**): COP (1.25 mg/mL), SCOP (9.75 µg/mL), SCOP-1 (9.75 µg/mL). Therapeutic mode (**G**–**L**): SCOP (9.75 µg/mL), SCOP-1 (9.75 µg/mL). Data are presented as mean ± SD (n = 3). ^*##*^*P* < 0.01 vs. blank control group; ^*^P < 0.05, ^**^P < 0.01 vs. virus group

To investigate the immunomodulatory potential of COP and its sulfated derivatives, we examined their effects on IAV-induced cytokine mRNA expression in A549 cells. As shown in Fig. 10, H1N1 infection significantly increased the mRNA level of *IL-6, IL-8, MCP-1, CCL-5, TNF-α*, and *TRAIL* (Fig. 10). Treatment with COP and SCOP under both direct virucidal and therapeutic modes effectively suppressed the expression of these genes. These results suggested that both COP and its sulfated derivatives possess antiviral and anti-inflammatory activity in A549 cells, with the sulfated derivatives exhibiting significantly enhanced efficacy under therapeutic mode.

Influenza A virus infection activates the NF-κB pathway and induces the release of large amounts of pro-inflammatory cytokines, forming a "cytokine storm" that exacerbates lung injury [41]. Our results indicate that Codonopsis polysaccharides and their sulfated derivatives may attenuate virus-induced inflammatory responses by inhibiting NF-κB activation. The sulfated derivatives, especially SCOP, effectively suppressed cytokine expression even under therapeutic mode, whereas unmodified COP showed no obvious effect in this setting, further confirming that sulfation modification enhances the anti-inflammatory activity of these compounds [11].

#### Effects of compounds on NF-κB signaling pathway protein expression under different modes of administration

**Fig. 11:**
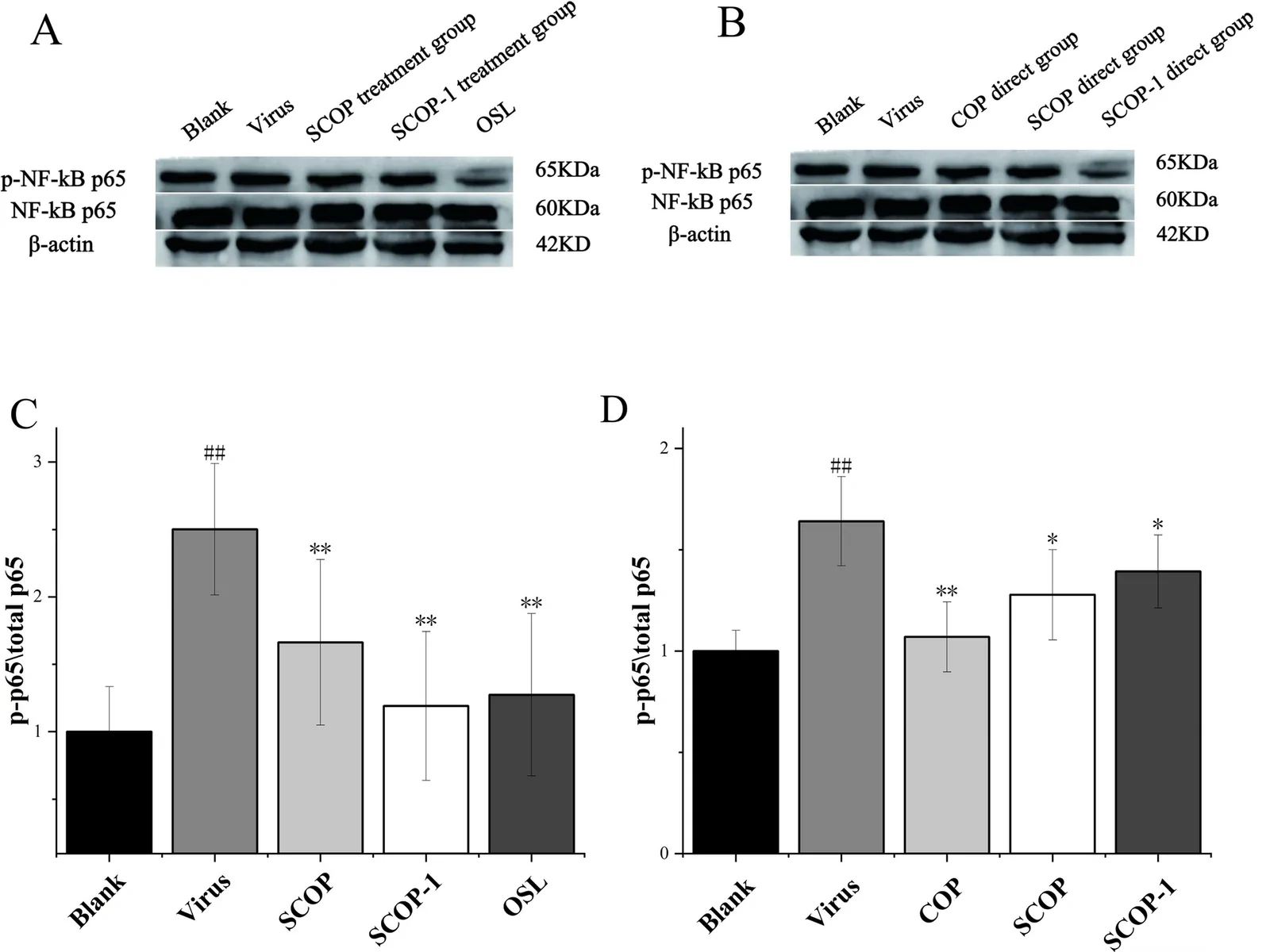
Effects of compounds on the expression of key proteins in NF-κB signaling pathway. Therapeutic mode (**A**, **B**): SCOP (9.75 µg/mL), SCOP‑1 (9.75 µg/mL). Direct virucidal mode (**C**, **D**): COP (1.25 mg/mL), SCOP (9.75 µg/mL), SCOP‑1 (9.75 µg/mL). **A**, **C** Representative Western blot images; **B**, **D** Quantitative analysis of p-p65/total p65 ratio. Data are presented as mean ± SD (n = 3). ^*##*^*P* < 0.01 vs. blank control group; ^*^P < 0.05, ^**^P < 0.01 vs. virus group

As illustrated in Fig. 11, the phosphorylation of p65, a key marker of NF-κB activation, was differentially inhibited depending on the compound and administration mode. Under the direct virucidal mode, COP and SCOP significantly reduced p‑NF‑κB p65 expression, while SCOP‑1 had no significant effect. Under the therapeutic mode, both SCOP and SCOP‑1 significantly reduced p‑NF‑κB p65 expression. These results indicate that sulfation enhances the ability of Codonopsis polysaccharides to inhibit the NF‑κB pathway, particularly under therapeutic mode [29].

The superior activity of SCOP (DS = 1.792) over SCOP‑1 (DS = 1.403) suggests that the degree of substitution may affect antiviral potency in sulfated derivatives [10]. SCOP‑1 had no significant effect on p‑NF‑κB p65 under direct virucidal mode but did show inhibition under therapeutic mode, suggesting that the mechanism of action may differ among administration modes. In the direct virucidal mode, the compound may first bind to viral particles, potentially altering its conformation or consuming some active molecules, thereby reducing its direct inhibition of the NF‑κB pathway in host cells; in the therapeutic mode, the compound acts directly on already infected cells and may enter cells more effectively to exert anti‑inflammatory effects. Taken together, the downregulate of pro‑inflammatory cytokines by COP, SCOP and SCOP‑1 is likely mediated through inhibition of NF‑κB p65 phosphorylation, indicating that the anti‑inflammatory effect of these polysaccharides depends at least in part on suppressing the NF‑κB signaling axis, a central driver of influenza‑induced cytokine storm [14].

## Conclusion

In conclusion, COP and its sulfated derivatives, SCOP and SCOP‑1, exhibit potent anti-influenza A (H1N1) activity in vitro. These compounds inhibit viral replication at an early stage (0–4 h), reduce virus-induced apoptosis, downregulate pro-inflammatory cytokines, and suppress inflammatory responses through blockade of the NF-κB pathway. Notably, sulfation modification significantly enhances these antiviral effects, positioning sulfated Codonopsis polysaccharides as promising candidates for novel anti-influenza therapeutics.

## Supplementary Information

Supplementary material 1.

## Data Availability

The data that support the findings of this study are available from the corresponding author upon reasonable request.
