# Supplementary material for: Structural characterization and antiviral activity of polysaccharide from Codonopsis pilosula
Source: Nat Prod Bioprospect. 2026 Oct 1;16(1):90. doi: 10.1007/s13659-026-00649-5 (PMC13627515; doi:10.1007/s13659-026-00649-5)
Supplement: Supplementary file 1 [file 13659_2026_649_MOESM1_ESM.docx]

Supplementary Information(SI)

**Structural characterization and antiviral activity of polysaccharide from *Codonopsis pilosula***

Supplementary Table

| **Position** | ***δ*_C_**  **(ppm) \[D_2_O]** | ***δ*_H_**  **(ppm) \[D_2_O]** | **Multiplicity J**  **(Hz)** | ***δ*_H_**  **(ppm) \[DMSO-*d*_6_]** | **Multiplicity J**  **(Hz)** | **Assignment** |
| --- | --- | --- | --- | --- | --- | --- |
| C-1 / H-1a | 62 | 3.76 | d, 10.0 | - | - | CH₂ |
| H-1b |  | 3.6 | m | - | - | CH₂ |
| C-2 | 103.1 | - | - | - | - | Anomeric C (quaternary) |
| C-3 / H-3 | 76.8 | 4.09 | d, 8.4 | - | - | CH |
| 3-OH |  | - | - | 4.75 | d, 5.6 | OH |
| C-4 / H-4 | 74.1 | 3.94 | t, 8.0 | - | - | CH |
| 4-OH |  | - | - | 5.18 | d, 4.4 | OH |
| C-5 / H-5 | 80.9 | 3.69 | m | - | - | CH |
| C-6 / H-6a | 60.7 | 3.69 | m | - | - | CH₂ |
| H-6b |  | 3.6 | m | - | - | CH₂ |
| 6-OH |  | - | - | 4.62 | s | OH |

Table S1 ^1^H and ^13^C NMR spectroscopic data for COP-1 in D_2_O and DMSO-*d*_6_

| **Correlation Type** | **Proton** | **Correlated Carbon/Proton** | **Structural Evidence** |
| --- | --- | --- | --- |
| HMBC | 3-OH (*δ*_H_ 4.75) | C-2 (*δ*_C_ 103.1), C-3 (*δ*_C_ 76.8), C-4 (*δ*_C_ 74.1) | Confirms OH-3 position |
| HMBC | 4-OH (*δ*_H_ 5.18) | C-3 (*δ*_C_ 76.8), C-4 (*δ*_C_ 74.1), C-5 (*δ*_C_ 80.9) | Confirms OH-4 position |
| HMBC | 6-OH (*δ*_H_ 4.62) | C-5 (*δ*_C_ 80.9), C-6 (*δ*_C_ 60.7) | Confirms OH-6 position |
| COSY | 6-OH (δ_H_ 4.62) | H-6 (*δ*_H_ 3.60, 3.69) | Confirms OH-6 connectivity |
| COSY | H-6 | H-5 (*δ*_H_ 3.69) | H-5/H-6 coupling |
| COSY | H-5 | H-4 (*δ*_H_ 3.94) | H-4/H-5 coupling |
| COSY | H-4 | H-3 (*δ*_H_ 4.09) | H-3/H-4 coupling |

Table S2 Key HMBC and COSY correlations for COP-1 in DMSO-*d*_6_.
